# Effect of Process Parameters, Protectants and Carrier Materials on the Survival of Yeast Cells during Fluidized Bed Granulation for Tableting

**DOI:** 10.3390/pharmaceutics15030884

**Published:** 2023-03-09

**Authors:** Karl Vorländer, Lukas Bahlmann, Arno Kwade, Jan Henrik Finke, Ingo Kampen

**Affiliations:** 1 Institute for Particle Technology, Technische Universität Braunschweig, Volkmaroder Straße 5, 38104 Braunschweig, Germany; lukas.bahlmann@tu-braunschweig.de (L.B.); a.kwade@tu-braunschweig.de (A.K.); jan.finke@tu-braunschweig.de (J.H.F.); i.kampen@tu-braunschweig.de (I.K.); 2 Center of Pharmaceutical Engineering (PVZ), Technische Universität Braunschweig, Franz-Liszt-Straße 35A, 38106 Braunschweig, Germany

**Keywords:** drying, fluidized bed spray granulation, probiotics, *Saccharomyces cerevisiae*, protective additives, direct compression excipients, temperature, moisture content

## Abstract

The administration of living microorganisms is of special interest, with regard to probiotic microorganisms providing health benefits to the patient. Effective dosage forms require the preservation of microbial viability until administration. Storage stability can be improved by drying, and the tablet is an especially attractive final solid dosage form due to its ease of administration and its good patient compliance. In this study, drying of the yeast *Saccharomyces cerevisiae* via fluidized bed spray granulation is investigated, as the probiotic *Saccharomyces boulardii* is a variety of it. Fluidized bed granulation enables faster drying than lyophilization on the one hand and lower temperatures than spray drying on the other hand, which are the two predominantly used techniques for life-sustaining drying of microorganisms. Yeast cell suspensions enriched with protective additives were sprayed onto the carrier particles of common tableting excipients, namely, dicalcium phosphate (DCP), lactose (LAC) and microcrystalline cellulose (MCC). Different protectants, such as mono-, di-, oligo- and polysaccharides, but also skimmed milk powder and one alditol, were tested; as they themselves, or chemically similar molecules, are known from other drying technologies to stabilize biological structures such as cell membranes, and thus, improve survival during dehydration. With the combined use of trehalose and skimmed milk powder, survival rates were 300 times higher than without the use of protective additives. In addition to these formulation aspects, the influence of process parameters such as inlet temperature and spray rate were considered. The granulated products were characterized regarding their particle size distribution, moisture content and the viability of the yeast cells. It has been shown that thermal stress on the microorganisms is especially critical, which can be reduced, for example, by reducing the inlet temperature or increasing the spray rate; however, formulation parameters such as cell concentration also influenced survival. The results were used to specify the influencing factors on the survival of microorganisms during fluidized bed granulation and to derive their linkages. Granules based on the three different carrier materials were tableted and the survival of the microorganisms was evaluated and linked to the tablet tensile strength achieved. Using LAC enabled the highest survival of the microorganisms throughout the considered process chain.

## 1. Introduction

Probiotic microorganisms are microorganisms that confer health benefits to the patient if they are administered in adequate amounts and in a viable form [1]. The mechanisms of action are diverse and range from competition with pathogens for nutrients to the formation of bacteriocins, which can act selectively on individual strains or similarly to broad-spectrum antibiotics [2]. However, as the viability of the microorganisms is essential, dosage forms preserving the probiotic’s viability during production, storage until administration, and reaching the target site after intake, are required. To allow sufficient storage stability, the microorganisms must be converted to a temporarily inactive state, either by freezing or drying. In order to maintain long-term viability in the dry state, suitable packaging of the final products is required, which is impermeable to moisture and oxygen, because these two parameters in particular, together with the storage temperature, affect the shelf life of probiotic products [3]. Therefore, single use formats such as sachets or blistered tablets are preferred to bulk packaging.

Freezing living cells so that they maintain their viability is critical, and the process and formulation must be adapted to avoid lethal conditions such as toxic increases in concentration during extracellular ice formation or the formation of intracellular ice crystals that can destroy the cell membrane [4,5,6]. In addition to these hurdles, permanent storage below the melting point is also necessary, which is why this type of preservation is avoided for probiotic preparations and instead the aim is to dry them as gently as possible. This also enables further processing into tablets as the final dosage form. Tablets have some major advantages, such as easy administration of exact doses, masking of any bad taste and odor of the microorganisms, and thus generally high patient acceptance. Therefore, this is often the delivery of choice for probiotics, even though compaction into tablets is a critical step for microbial viability [7,8,9].

While freeze-drying is generally considered to be particularly gentle, the aforementioned challenges of freezing are counterpart to the already critical impact of dehydration. In addition, the process is slow and investment and energy intensive. Although lyophilized yeast cells were also already tableted, an additional upstream comminution step was necessary, which was accompanied by an additional reduction in viability [9]. Furthermore, the mechanical strength of the tablets was comparatively low, so high compaction stresses had to be applied, which negatively affected the viability of the cells in the tablets.

Convective drying processes such as spray drying enable significantly faster drying, however considerably higher temperatures are used for this process. On the one hand, this has the advantage that the microorganisms are not damaged by a freezing step, but there is a risk of thermal denaturation. One possibility for drying at a lower temperature is the use of a fluid bed processor, which is used in this study for drying of the yeast *Saccharomyces cerevisiae* via spray granulation. This microorganism was chosen because the probiotic yeast *Saccharomyces cerevisiae var. boulardii* belongs to this specie [10] and it is assumed that the basic dependencies on process parameters are identical for both strains, even if the concrete survival rates do not necessarily have to match exactly due to strain specific tolerances. Furthermore, *Saccharomyces cerevisiae* was more easily accessible in the required quantities. In general, fluidized bed granulation requires some carriers or matrix builders onto which the granulation fluid is sprayed and to which the cells can adhere in the present case. Although fluidized bed granulation has been used for the life-sustaining drying of microorganisms for more than 30 years [11], it has been used predominantly in the food sector, for example for drying starter cultures. Furthermore, wheat and rye flour or sweet whey powder, for example, were used as carrier materials [11,12], i.e., carrier materials that would not be acceptable for pharmaceutical applications, especially if subsequent tableting was intended. Therefore, the extent to which typical carrier materials from the pharmaceutical environment are also suitable for the life-sustaining granulation of microorganisms will be investigated in this work; as such, dicalcium phosphate (DCP), lactose (LAC) and microcrystalline cellulose (MCC), carrier materials with high importance in the field of tableting, are used.

During granulation, the carrier material is fluidized in the fluidized bed processor and the cell suspension is sprayed on these particles [6]. Depending on the process parameters, carrier particles are either coated with the cells [13,14] or the particles are granulated with cells [6,15] and, if necessary, with additional binders. For example, *Enterococcus faecium* M74 was coated on MCC pellets improving flowability compared to lyophilizates enabling proper further processability to solid dosages [16]. Normally, the coating procedure requires longer process time as the spray rate has to be low enough to prevent agglomeration. However, the long process time could be critical as the cells are exposed to potentially harmful process temperatures for a longer time, which is why granulation is the focus of this study. In addition, the tabletability of such granules is generally better than that of, for example, coated MCC pellets [17]. The influence of various process and formulation parameters is investigated. These include the type and amount of protective additives in the spray suspension, the spray rate, inlet temperature, post-drying time and cell concentration. The findings will be used to derive process conditions that enable improved survival during granulation.

Basically, tableting of dried microorganisms is challenging due to the high compression stresses that have to be applied. In general, the viability of microorganisms decreases with increasing compression stress [7,8,9,18,19,20,21,22,23,24,25,26,27,28]. However, previous data on the survival of microorganisms during tableting exist predominantly for lyophilized microorganisms [9,18,19,20,21,22,27,29,30,31,32,33,34,35,36,37,38] and are hardly available for fluidized bed granulated ones; however, at least some data are available for wet granulated and, e.g., oven-dried microorganisms [7,8,23,24,25,39,40]. Considering, especially, the different product structures (physical mixture vs. granules and the typically high porosity and fragility of lyophilized products), the survival during tableting needs to also be addressed for granulated microorganisms. The lower survival when higher stresses were applied was attributed to increasing mechanical stress and damage to essential cell components, especially the cell membrane. This is another reason why yeast cells were used for the studies instead of bacteria. It is assumed that the larger size of the yeast cells is critical regarding the mechanical stresses since pressure and shear have an increased lethal effect on large cells [7,24]. It is therefore assumed that mechanical damage is of higher relevance when tableting using yeast cells than when using much smaller bacterial cells, and that the mechanisms can be better understood.

However, how critical tableting is to the cells is formulation-dependent. That is why, regardless of how high the survival after granulation, in this work, granules based on three different tableting excipients will be used for compaction and the microbiological properties, as well as the tensile strength of the tablets, will be determined.

## 2. Materials and Methods

### 2.1. Cell Suspension

Baker’s yeast *Saccharomyces cerevisiae* (Deutsche Hefewerke, Nürnberg, Germany) was used for all experiments. The dry mass content of the yeast cells was 29.7 ± 0.6%. Depending on the experiment, 100, 150 or 200 g L^−1^ of yeast cell dry mass were suspended in water or solutions of different protective additives and incubated for 1 h at room temperature while mixing with a magnetic stirrer prior to granulation. This allows equilibration between microorganisms and protectants and is typical prior to freeze-drying [41,42] and has also been applied before fluidized bed drying [43,44]. Afterwards, the suspension was directly used for granulation. To prevent cell growth (division of cells) and metabolic activity (e.g., breakdown of sugars under formation of carbon dioxide) of the suspended cells during granulation (duration of up to 60 min)**,** the suspension was stored on ice during the granulation process. Otherwise, the formation of carbon dioxide, especially in the tubes, would have negatively affected the pumping of the suspension. The initial viability of the suspended cells was determined (see Section 2.4.2) after the incubation at the beginning of the granulation, as well as at the end of the granulation process, and the mean value of both measurements was calculated. However, as expected, due to the duplication time of the yeast of 1.4 h under optimal conditions (bioreactor cultivation in yeast-peptone-dextrose medium, 30 °C, data not shown) and the suboptimal conditions, especially due to the cooling, the two values did not differ significantly.

### 2.2. Granulation Procedure

Dicalcium phosphate (DCP, DI-CAFOS A150, kindly provided by Chemische Fabrik Budenheim KG, Budenheim, Germany), lactose (LAC, Granulac 70, kindly provided by MEGGLE GmbH & Co. KG, Wasserburg am Inn, Germany) and microcrystalline cellulose (MCC, Vivapur 102, kindly provided by J. Rettenmaier & Söhne GmbH + Co. KG, Rosenberg, Germany) were used as carrier materials for the granulation. The fluidized bed device MiniGlatt (Glatt GmbH, Binzen, Germany) with a top spray configuration and maximal nozzle height was used for the experiments. The cell suspension was pumped with a peristaltic pump (Watson-Marlow 505U, Watson-Marlow Fluid Technology Group, Falmouth (Cornwell), United Kingdom) through a two-fluid nozzle (0.5 mm diameter). The air pressure of the nozzle was always 0.2 bar higher than the process air pressure. Depending on the parameters studied, a carrier mass of up to 200 g was granulated. Prior to the granulation, the carrier particles were dried for 10 min. The process air pressure, and by this the volume flow, needed to be adjusted depending on the carrier material to achieve a suitably fluidized bed. The granules were characterized regarding their moisture content and the survival of the yeast cells and findings, as described below, were used to improve the process (see Section 2.2.7).

#### 2.2.1. Screening of Protective Additives

Many protective additives are known to not only increase cell survival in one type of drying process, but can also be beneficial in other drying processes [6]. Different additives were known from previous investigations to protect *Saccharomyces cerevisiae* during freeze-drying [9]. Based on these experiments, different protectants, such as mono-, di-, oligo- and polysaccharides, but also skimmed milk powder and one alditol, were tested regarding their suitability to preserve the viability of the yeast cells during fluidized bed spray granulation. For this, 100 g L^−1^ of yeast cell dry mass was mixed as described above with suspensions or solutions, containing 100 g L^−1^ of dextran 20, d(−)-fructose, d(+)-glucose monohydrate, corn starch, skimmed milk powder, d(+)-sucrose, d-sorbitol, d(+)-xylose (all from Carl Roth GmbH + Co. KG, Karlsruhe, Germany), inulin (Spinnrad GmbH, Bad Segeberg, Germany), α-lactose monohydrate (kindly provided by Meggle GmbH & Co. KG. Wasserburg am Inn, Germany), maltodextrin 6 (Berco Arzneimittel Gottfried Herzberg GmbH, Kleve, Germany) or trehalose dihydrate (FormMed HealthCare AG, Frankfurt am Main, Germany), respectively. In addition, combinations of 50 g L^−1^ of trehalose dihydrate and 50 g L^−1^ as well as 100 g L^−1^ of skimmed milk powder were tested. In total, 50 g of MCC was used as the carrier material, the inlet temperature was 50 °C, the yeast cell concentration was 100 g L^−1^ (dry mass), spray rate 1.76 mL min^−1^, granulation time 15 min and the subsequent drying time was 5 min (Table 1).

#### 2.2.2. Variation of Carrier Material

In another series of experiments, different carrier materials were used to evaluate how they affect the survival of microorganisms during granulation. For this purpose, 200 g of DCP, LAC or MCC was granulated with a cell suspension comprising a dry mass content of yeast of 100 g L^−1^, and 100 g L^−1^ of trehalose dihydrate and 50 g L^−1^ of skimmed milk powder as protective additives (Table 1). The inlet temperature of the process air was 50 °C for all batches; however, the volume flow was adjusted depending on the carrier material to achieve a suitable fluidized bed. Due to the increase in particle size during granulation, it was necessary to increase the process air pressure and thus the volume flow rate of the drying air in order to be able to maintain the fluidized bed. The spray rate was 1.76 mL min^−1^ and granulation was performed for 60 min. Subsequently, the product was dried for an additional 10 min at the same inlet temperature.

#### 2.2.3. Variation of Spray Rate and Process Time

Using 200 g of LAC as a carrier material, the granulation was repeated as described in Section 2.2.2, however, the spray rate was increased to allow shorter process times with the same loading of yeast cells onto the carriers (Table 1). With spray rates of 2.64 and 5.28 mL min^−1^, it was possible to spray the same amount of cell suspension in 40 and 20 min, respectively. Again, it was necessary to adjust the process air pressure during the granulation depending on the size of the granules, which increased faster for higher spray rates. In this study, stated loadings $\chi_{\mathrm{CDW},\;\mathrm{granules}}$ were calculated on the basis of theoretical assumptions neglecting any (inhomogeneous) losses that may occur (powder, components of the cell suspension)

(1) $$\chi_{\mathrm{CDW},\;\mathrm{granules}}=\frac{m_{\mathrm{suspension},\;\mathrm{sprayed}}\cdot \chi_{\mathrm{CDW},\;\mathrm{suspension}}}{m_{\mathrm{granules}}\cdot \left(1-\varphi_{\mathrm{granules}}\right)}$$

with the used suspension mass $m_{\mathrm{suspension},\;\mathrm{sprayed}}$, its proportion of cell dry weight $\chi_{\mathrm{CDW},\;\mathrm{suspension}}$, the obtained granules mass $m_{\mathrm{granules}}$ and the moisture content of the granules $\varphi_{\mathrm{granules}}$.

#### 2.2.4. Variation of Process Temperature

The influence of the process temperature on the survival of the yeast cells was investigated using 200 g of LAC as a carrier. The granulation was performed as described in Section 2.2.2. Deviating from this, inlet temperatures of 30, 40, 60 and 70 °C were used (Table 1). The process air pressure was kept as equal as possible for all temperatures.

#### 2.2.5. Variation of Final Drying Time

With only 50 g of MCC and 15 min spray time, the granulation was repeated four times and the process was stopped directly after stopping spraying or after 5, 10 and 30 min of subsequent drying, respectively. The inlet temperature was 50 °C throughout the whole process and the spray rate 1.76 mL min^−1^ (Table 1).

#### 2.2.6. Variation of Cell Concentration

In total, 50 g of MCC was granulated with a cell suspension containing, again, 100 g L^−1^ of trehalose dihydrate and 50 g L^−1^ of skimmed milk powder; however, this time not only 100 but also 50 and 200 g L^−1^ of yeast cell dry mass for 15 min (Table 1). Again, the spray rate was 1.76 mL min^−1^ and the inlet temperature was 50 °C, however, the subsequent drying was performed for only 5 min considering the results of the experiments in Section 2.2.5.

#### 2.2.7. Combination of Advantageous Process and Formulation Parameters

Process and formulation parameters for improving the process were derived from the findings of the previously described investigations. In total, 200 g of the carrier (all carrier materials were still considered) was granulated for 40 min at 50 °C with a spray rate of 2.64 mL min^−1^ (Table 1). The granulation suspension contained 200 g L^−1^ of yeast cell dry mass and 100 g L^−1^ of trehalose dihydrate and skimmed milk powder each. No subsequent drying was carried out.

### 2.3. Tableting

A compaction simulator (Styl’One evolution, Medelpharm, Beynost, France) equipped with EUR-D tools with a diameter of 11.28 mm was used to produce cylindrical tablets with a mass of 450 mg. Compression stresses in a range of 50–400 MPa were applied with a generic, symmetrical compression profile, with a given pressure increase/decrease time of 90 ms and a dwell time of 20 ms. Granules made of LAC and DCP were mixed for 2 min at 49 min^−1^ in a 3D shaker mixer (TURBULA, Willi A. Bachofen AG, Muttenz, Switzerland) with 0.5 wt.-% of magnesium stearate as a lubricant to reduce the ejection forces. For each formulation and applied compression stress, 3 tablets were used for quantification of microbiological survival (Section 2.4.2) 2 h after compaction.

### 2.4. Analysis of Granules and Tablets

#### 2.4.1. Moisture Content

The moisture content of the granulated products was determined as mass loss during drying for 24 h at 80 °C in a drying oven (Heratherm OGS100, Thermo Electron LED GmbH, Langenselbold, Germany).

#### 2.4.2. Survival

The viability of the processed and unprocessed yeast cells was investigated via the determination of colony forming units (CFU). Samples of the sprayed suspension, for reference purpose (mean of determination at the beginning and end of granulation), were serially diluted in a phosphate buffered saline (PBS) solution (1.6 g L^−1^ NaCl, 0.04 g L^−1^ KCl, 0.284 g L^−1^ Na_2_HPO_4_, 0.054 g L^−1^ KH_2_PO_4_, pH 7.4; Sigma-Aldrich Chemie GmbH, München, Germany) until they reached a suitable concentration. Samples of granules and tablets were given to such a volume of PBS that a concentration of 100 g L^−1^ cell dry mass was obtained. This was followed by magnetically stirring all of the samples for 60 min, guaranteeing the same treatment for all samples independent of the disintegration time, which differed for the tested formulations, and applied compression stress (longest disintegration time determined according to Ph. Eur. for tablets with MCC and an applied compression stress of 400 MPa but still < 30 min). These suspensions were serially diluted with PBS as well, until they reached a suitable concentration. It is important to always follow this procedure exactly the same way (concentration as well as time), since different parameters of rehydration have an influence on survival [43,44,45], and otherwise, could overlay the actual effects of the drying parameters studied. Diluted suspensions were spread onto yeast extract peptone dextrose agar plates in triplicates (10 g L^−1^ of yeast extract, 20 g L^−1^ of peptone ex casein, 22 g L^−1^ of glucose monohydrate and 15 g L^−1^ of Agar-Agar Kobe 1; all from Carl Roth GmbH + Co. KG, Karlsruhe, Germany) [9]. After incubation for approx. 30 h at 30 °C, the colonies were counted manually. Absolute viability was calculated as the CFUs per gram of cell dry weight (CDW) (Equation (2)). Survival rates were calculated as the percentage of remaining viability after being processed (Equation (3)).

(2) $$\mathrm{viability}\;\left[\mathrm{CFU}\;{g\;}_{\mathrm{CDW}}^{-1}\right]=\frac{\mathrm{count}\;\mathrm{of}\;\mathrm{colonies}\;\left[\mathrm{CFU}\right]}{\mathrm{plated}\;\mathrm{concentration}\;\left[g_{\mathrm{CDW}}{\;L}^{-1}\right]\;\cdot \;\mathrm{plated}\;\mathrm{volume}\;[L]}$$

(3) $$\mathrm{survival}\;\mathrm{rate}\;\left[\%\right]=\frac{\mathrm{viability}\;\mathrm{after}\;\mathrm{granulation}\;\mathrm{or}\;\mathrm{compaction}\;\left[\mathrm{CFU}\;{g\;}_{\mathrm{CDW}}^{-1}\right]}{\mathrm{viability}\;\mathrm{of}\;\mathrm{fresh}\;\mathrm{cells}\;\left[\mathrm{CFU}\;{g\;}_{\mathrm{CDW}}^{-1}\right]}$$

#### 2.4.3. Particle Size

The particle size of the carriers DCP, LAC and MCC, as well as the corresponding granules, was analyzed via dynamic image analysis with a QICPIC (Sympatec GmbH, Clausthal-Zellerfeld, Germany) equipped with a GRADIS dispersing unit to ensure a gentle dispersion in free fall, and a VIBRI dosing unit to ensure a constant sample mass-flow. The analysis was performed three times, with at least 100,000 particles analyzed each time.

### 2.5. Density of Carrier Materials and Granules

True density of the formulations was determined by helium gas pycnometry (Ultrapyc 1200e, Quantachrome Instruments, Boynton Beach, FL, USA).

### 2.6. Tensile Strength of Tablets

Tablet height *h*, diameter *d*, and breaking force *F* were measured 24 h after production according to the European Pharmacopeia (Ph. Eur. 9.2 2.9.8) using a manual tablet tester (MultiTest 50, Sotax AG, Aesch, Switzerland) to calculate the tablet tensile strength $\sigma_{t}$ [46].

(4) $$\sigma_{t}=\frac{2\cdot F}{\pi \cdot d\cdot h}$$

### 2.7. Scanning Electrone Microscopy

Samples were sputtered with platinum (LEICA EM ACE600, Leica Microsystems GmbH, Wetzlar, Germany) prior to imaging with a Helios G4 CX (FEI, Hilsboro, OR, USA).

### 2.8. Statistical Analysis

For statistical analysis of the results, an analysis of variance function (ANOVA) implemented in OriginPro (Version 2023, OriginLab Corporation, Northampton, MA, USA) was used. Data points in the graphs that do not share a letter are significantly different (significance level of α = 0.05).

## 3. Results and Discussion

### 3.1. Influence of Protective Additives

**Figure 1:**
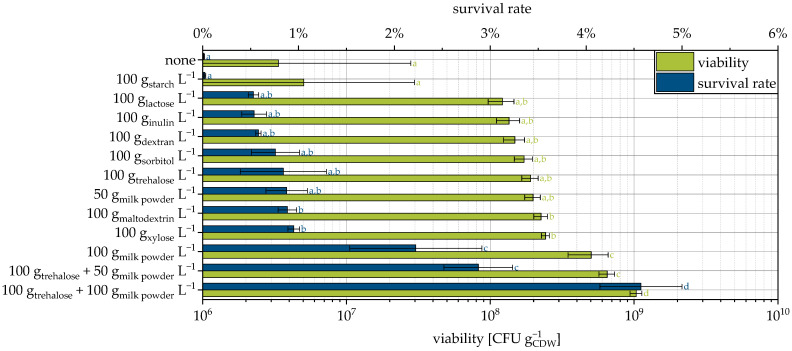
Viability and survival rate of yeast cells granulated with different protectants and MCC as the carrier. Granulation with fructose, glucose, and sucrose were not possible due to excessive gas formation of the cell suspension. Data points that do not share a letter are significantly different (significance level α = 0.05).

When the yeast cells were suspended in water and used as a granulation liquid, the survival rate was very low at about 0.015% (Figure 1). Testing of different protective additives allowed a significant increase in the survival rate (up to 300 times). The highest survival rates were observed with a combination of trehalose and skimmed milk powder. Saccharides and skimmed milk are known to enhance survival during fluidized bed drying [6]. However, at just 4.5%, the survival rate here is also very low.

Freeze-drying of the same yeast strain has enabled survival rates up to 42.9% [9], and fluidized bed drying of *Saccharomyces cerevisiae* pellets (another strain), without protectants, has enabled survival rates of up to and more than 90% [47,48]. In contrast, only significantly lower survival rates are achieved in the present study. Why similarly high survival rates are not achieved here has not yet been found out. In general, during drying, water is removed from the cell membrane, bringing the head groups of the phospholipids closer together [49]. As a result, the alkyl chains also move closer together and van der Waals interactions increase what, in turn, causes a transition of the membrane from a liquid-crystalline to a gel state [50]. In the course of rehydration and by this the reverse change from the gel phase back to the liquid-crystalline phase, packaging defects and leakages in the cytoplasmic membrane may occur [51,52]. Sugars such as trehalose can prevent the transition to the gel phase during drying, so that the membrane is also in the liquid-crystalline state even if dry, and the critical reverse phase change during rehydration is avoided [50]. In this water replacement theory, hydrogen bonds between sugar molecules and phospholipid head groups are assumed to preserve the distance of the phospholipids [49,50,51].

In addition, sugars are thought to protect membranes by entrapping the water molecules surrounding the phospholipid head groups (also known as the preferential hydration or exclusion hypothesis) [53,54,55]. A third hypothesis is based on the low mobility of vitrified molecules due to high glass transition temperatures (also known as the viscosity hypothesis) [54]. The structure is mechanically preserved preventing denaturing molecule movements. In addition to membrane stabilization, the structure and function of essential proteins must also be preserved. In analogy to the phase preservation of membranes, sugars enable proteins to be stabilized in a conformation that resembles the natural hydrated form [50]. This prevents incorrect refolding of the protein’s tertiary structure during rehydration. The same applies to the hypothesis of preferential hydration and vitrification.

In order to understand the effect of the milk powder, it is necessary to consider another mechanism that can lead to a loss of viability: Membrane associated proteins are easier to remove from the membrane after the procedure of drying and rehydration [56]. Therefore, the cells lose, among others, a part of the permease system and inhibitors of degrading enzymes. This causes the activation of these and, consequently, a loss of viability. It is assumed that the proteins of the milk powder form a protective layer around the cells and thus reduce the loss of cellular proteins, enhancing the survival rate [56]. In addition, the contained lactose itself acts as a protective substance (Figure 1). The same models as previously described for saccharides apply here. It is precisely the interplay of the individual mechanisms of different protectors that appears to be particularly advantageous, even if the survival rate still has room for improvement.

Possibly, the process-related recurrent wetting of the cells is critical, since the phase change of the membrane occurs several times and is, probably, mostly incomplete, resulting in irreversible structural changes. However, temperatures above the critical limit should not have occurred here. In principle, of course, each strain has specific tolerances. The strain used in this study is optimized for use as fresh compressed yeast with only approx. 30% dry weight for baking and not for drying.

**Figure 2:**
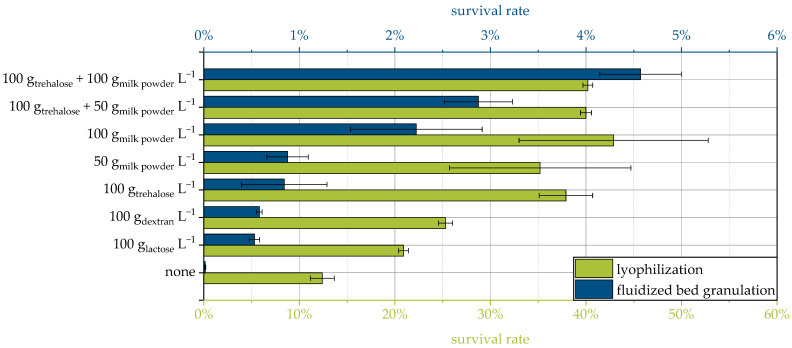
Comparison of the survival rates of yeast cells dried via fluidized bed granulation and lyophilization for selected protective additives. Results for lyophilization are from a previous study [9].

Some of the protective additives in this study have already been used for lyophilization of the same yeast strain in a previous study [9]. Although the survival rates span a different range and, relatively speaking, the influence of the protective additives is smaller for lyophilization, in principle, a similar ranking of the protective effect is obtained for both drying methods (Figure 2). This supports the mechanistic explanation given above, which is not necessarily limited to a specific drying method. For further conclusions, more different protective additives for drying the same strain would have to be compared with both processes and, ideally, others, such as spray drying.

**Figure 3:**
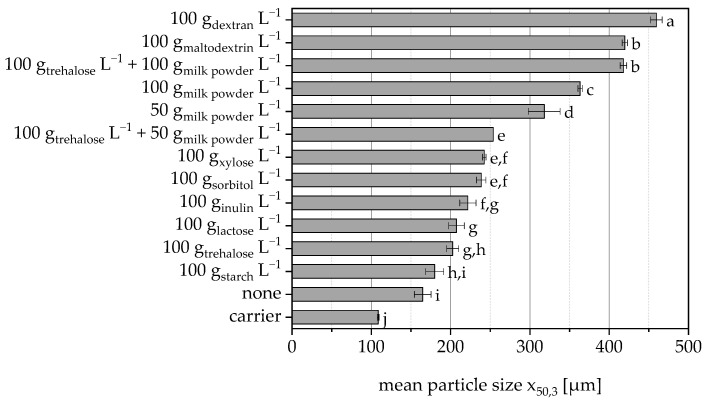
Mean particle size of granules based on MCC when using different protective substances in the sprayed cell suspension. Data points that do not share a letter are significantly different (significance level α = 0.05).

The additives used to protect the cells also act as binders during granulation. The effectiveness of these additives varied in terms of their ability to enable bond formation, and depending on the protective additive used or the amount of it, granules with different average particle sizes were obtained (Figure 3). The cell suspension without protective additive also acted as a binder and led to a significant increase in particle size. Dextran, maltodextrin and milk powder (also in combination with trehalose) showed the best binding properties and thus particle size of the granules (please note the different amounts of additives used).

### 3.2. Influence of Carrier Material

**Figure 4:**
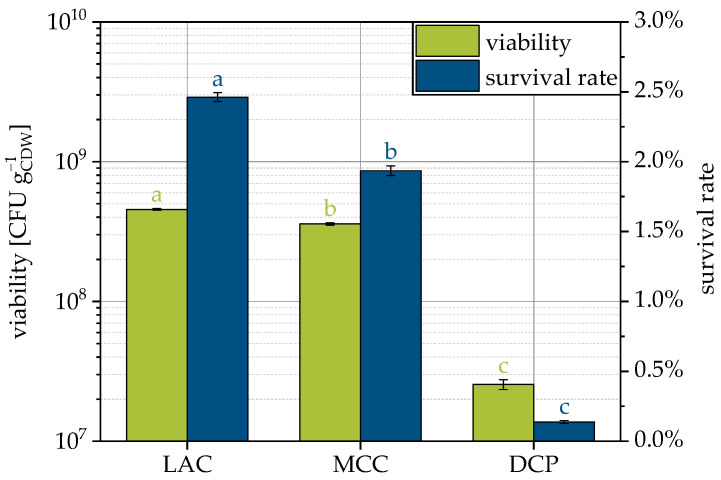
Viability and survival rate of yeast cells for granulation with different carrier materials. Data points that do not share a letter are significantly different (significance level α = 0.05).

Yeast suspension enriched with trehalose and skimmed milk as protectants was sprayed on typical tableting excipients DCP, LAC and MCC to produce granules while maintaining the viability of the microorganisms. Theoretically, the same cell number per final product mass was applied. Granules containing viable yeast cells could be produced with all three carrier materials (Figure 4). However, the viability calculated as CFUs related to the cell dry weight (CDW) and corresponding survival rates were low compared to lyophilization of this strain, where a 40% survival rate could be achieved [9]. The lowest survival rate of only 0.1% was observed for DCP followed by MCC with approx. 2.0% and LAC with approx. 2.5%. Despite the overall low survival rates, these rates differ significantly for the carriers considered. The carrier materials used are generally not considered cytotoxic. In separate experiments, it was measured that suspending DCP in water reduced the pH to 4.5. Such behavior was not observed with the other compounds. Nevertheless, it is not assumed that the pH shift caused the lowest survival rate with DCP, since optimal pH values for yeast cells vary from 4.5 to 6.0 [57]. Rather, it appears to be a secondary influence of the substrate. DCP is an inorganic material with almost twice the density of the organic carriers (see Table 2). In order to fluidize the heavier DCP particles, higher process air pressures were necessary, which was accompanied by higher drying air volume flows. If the drying gas flow rate is increased but the inlet temperature and spray rate are kept constant, the specific thermal energy input increases. This results in higher bed and outlet temperatures and is the cause of lower survival rates due to increased thermal stress on the cells.

### 3.3. Influence of Spray Rate

Different spray rates were tested to evaluate the influence of different product/outlet temperatures. For this purpose, the granulation time was varied in such a way that, despite the different process times, the loading of the carrier particles with yeast cells was the same at the end. Due to the experimental setup, with always the same inlet temperature, the different spray rates resulted in different moisture conditions and thus product temperatures. These could not be measured in the MiniGlatt system, but the measurement of the outlet temperature allows a comparable interpretation. For the same inlet temperature of 50 °C, the outlet temperatures at spray rates of 1.76, 2.64 and 5.28 mL min^−1^ were approx. 32, 31.5 and 28.5 °C (measured after 5 min of granulation, no steady-state), respectively. However, the highest spray rate selected resulted in an over-wetting of the fluidized bed and the consequent collapse of the bed, as the energy input was insufficient for drying. Results from the other two spray rates indicate that higher spray rates are beneficial to yeast cell survival (2.46 ± 0.03% and 7.5 ± 0.1% for 1.76 and 2.64 mL min^−1^, respectively). The associated lower temperature in the process chamber and simultaneously shorter process time is assumed to be the cause of this since the thermal stresses are reduced in duration and intensity. However, higher spray rates were also associated with the formation of coarser granules. This required an increase in the process air pressure and thus in the drying gas volume flow. Despite the constant inlet temperature, this increases the energy input into the drying chamber. In the case considered here, however, the advantages of the lower temperature in the process chamber and the shorter process time seem to outweigh this effect. In principle, therefore, the highest possible spray rates should be used, although the interaction with other process parameters sets boundaries in this respect.

### 3.4. Influence of Process Temperature

**Figure 5:**
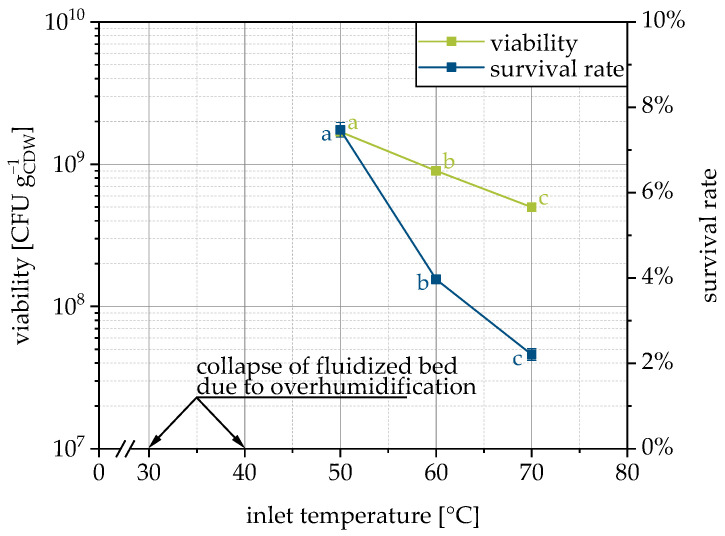
Dependency of viability and survival rate on inlet temperature for LAC as the carrier. Data points that do not share a letter are significantly different (significance level α = 0.05).

In order to consider the influence of the process temperature separately, the inlet temperature was varied in the range from 30–70 °C. The process pressures and thus the drying air volume flow was kept as equal as possible for all temperatures. As a consequence, the energy input at the two lowest temperatures was not sufficient for drying and the over-moistened fluidized bed collapsed (30 °C) or big lumps were formed (40 °C). Besides this, the survival of the yeast cells decreases with increasing temperature (inlet and thus also process chamber and outlet) (Figure 5), as expected and reported by other researchers [48,58] (please note that fluidized bed drying of yeast pellets and no granulation was performed).

Temperature-induced cell death of *Saccharomyces cerevisiae* occurs between 41–42 °C [59]. During the granulation process, the exhaust air temperature was always below 40 °C. Only during post-drying at 60 and 70 °C was this temperature level exceeded. It should be noted, however, that the inlet and outlet temperatures alone are not sufficient to quantify the temperature to which the microorganisms were exposed. In supplementary experiments, therefore, we attempted to also measure the product temperature in the fluidized bed. Unfortunately, no reliable conclusion can be made here regarding the question of whether the cells were effectively exposed to temperatures above the critical limit. Moist powder adhered to the temperature sensor and drying this powder caused the measured temperature to decrease significantly due to evaporative cooling. Simultaneously, drying reduced the adhesive forces, so the dried product detached from the temperature sensor, which was associated with a sudden rise in temperature until moist powder adhered again. Nevertheless, it can be assumed that at an inlet temperature of 50 °C and a corresponding outlet temperature of 28 °C, the cells have not exceeded the critical temperature of 41–42 °C. At an inlet temperature of 70 °C and an outlet temperature of 38 °C, the critical temperature may have been exceeded (perhaps only locally or temporarily). At the same time, the difference in survival rates at 50 and 70 °C is not as different as it is supposed to be if this temperature limit was reached. Moreover, the critical temperature was determined for cells in liquid media [59] and may not be transferred directly. In addition, the inactivation is time-dependent and reaching it for a short time does not inactivate all yeast cells immediately. Presumably, the drying of the wet granules here still causes sufficient evaporative cooling, so that a critical temperature limit was not exceeded for the yeast cells or only for a short time.

The increase in inlet temperature was also accompanied by a reduction in mean particle size x_50,3_, as expected. At inlet temperatures of 50, 60, and 70 °C, this was 705 ± 7, 580 ± 4 and 430 ± 16 µm, respectively. Due to the higher temperature, faster drying occurs, which is why fewer liquid bridges are formed, which dry and become stable solid bridges. Possibly, the granule size also has an influence on the survival rate of the microorganisms, if a greater proportion of them are shielded from the hot drying air inside large granules. Attempts to look at this in more detail by using different grades of MCC with different initial particle sizes failed, because the necessary process parameters were too different (especially the inlet air flow rate but also the spray rate) or no stable process was possible (very fine initial particles). Therefore, the observed survival rates could not be meaningfully related to the initial particle or measured granule size.

### 3.5. Influence of Final Drying

**Figure 6:**
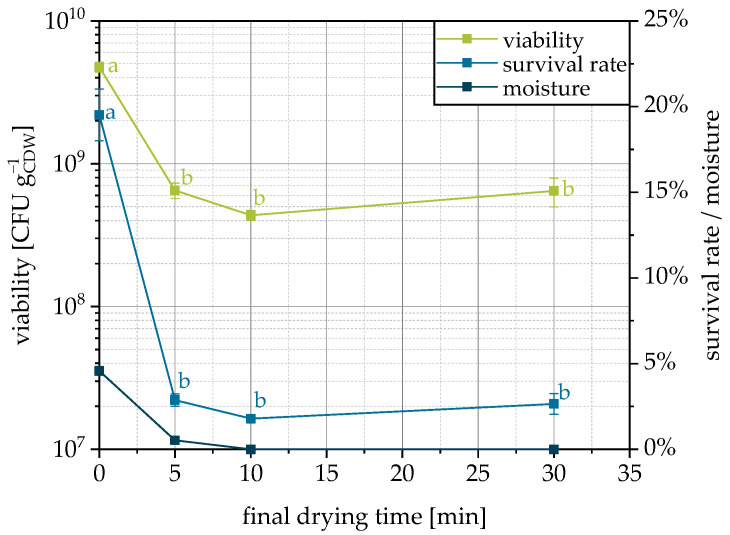
Viability, survival rate and moisture content of granules as related to the applied final drying time on MCC as a carrier. Data points that do not share a letter are significantly different (significance level α = 0.05).

If fluidization and, by this, drying is stopped directly with the end of spraying of the cell suspension and the product is removed, it has a significantly higher residual moisture compared to a post-drying of 5–30 min (Figure 6). After 5 min of fluidization and further drying after the end of granulation, the residual moisture is already significantly reduced, and after 10 min, the product is completely dried. However, this drying is accompanied by a decrease in the viability and survival rate. Two factors are responsible for this. First, the temperature in the fluidized bed increases when the moisture content decreases, since the energy is no longer spent on evaporating the water. This temperature rise can increase the denaturation processes and thus reduce the survival rate. Secondly, it is known that a certain residual moisture is beneficial to preserve the viability of dried microorganisms since irreversible structural changes occur when chemically bound water is removed [60,61]. For fluidized bed drying of active yeast pellets, it was shown that moisture content has the main influence and that the survival rates were equal for different inlet temperatures but with the same residual moisture [61,62]. As the viability decreases with decreasing moisture, residual moisture contents from 5–8% are typical for fluidized bed dried yeast pellets [61]. For improvement of the granulation process studied here, it can therefore be stated that no post-drying should take place, since the cells are already sufficiently dry for the conservation of viability, but further drying reduces survival.

### 3.6. Influence of Cell Concentration

**Figure 7:**
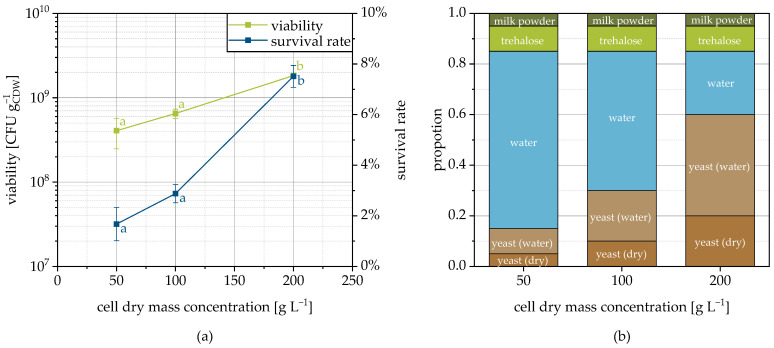
Viability and survival rate depending on the cell concentration of the sprayed suspension on MCC as the carrier (**a**) and the formulation composition (**b**). The amount of yeast (water) has to be understood as the cellular moisture content. Data points that do not share a letter are significantly different (significance level α = 0.05).

Increasing the cell concentration in the spray suspension resulted in an increase in the viability of the dried yeast cells (Figure 7a). This relationship was not observed during spray drying of *Lactobacillus rhamnosus* [63] but has already been observed in the freeze-drying of different bacterial strains and explained via mutual protection through shielding of the microorganisms [64]. The same correlation was also suspected for fluidized bed granulation, but could not be proven with any confidence due to the use of different strains with individual tolerances [44]. How, exactly, mutual protection occurs is not considered in the literature. In order to provide an explanation, it is necessary to take a closer look at the consequences of the higher cell concentration. On the one hand, this affects the viscosity of the cell suspension. The higher the cell concentration the higher the viscosity and the higher the average droplet size [65]. This influences the drying kinetics and maybe by this the microbial survival. Moreover, the number of cells on the surface of a droplet increases quadratically with cell concentration, whereas the number inside increases cubically. Therefore, proportionally fewer cells are directly exposed to the drying air (temperature and oxygen). If a part of the water of the cell suspension is substituted by cells, but absolutely the same amount of protective additives is used, the ratio of protective additive to cell decreases. Probably, this has a negative effect on the survival of the microorganisms especially at the granulate surface.

At the same time, the higher proportion of cells also implies an effectively higher protective additive concentration around the cells in the granulation liquid, which may explain the increased survival rate. Since yeast cells contain approximately 70% of water, the amount of free water was significantly reduced when the cell concentration changed from 50 to 200 g_CDW_ L^−1^ (Figure 7b) resulting in the higher effective concentrations (approx. 215, 270 and 600 $g_{\mathrm{protectants}}\;L_{\mathrm{free}\;\mathrm{water}}^{-1}$ for cell dry mass concentrations of 50, 100 and 200 g_CDW_ L^−1^, respectively). The higher protective additive concentrations in the continuous phase of the granulation fluid could, on the one hand, increase the uptake of protective additives by the cells and, on the other hand, also favor their partial dehydration, which is associated with increased temperature resistance [66]. It is possible that the lower concentrations of protectants used during spray drying of *L. rhamnosus* [63,67] contributed to the fact that a higher cell concentration did not confer a survival advantage for this case. In addition, there may be an influence of the strain, the protective substances [63] and the process. Please note, that the considerations presented are of a purely phenomenological nature and it is currently not yet possible to prove by experimental data whether and to what extent they actually come to bear, which will be addressed in future studies.

At this point, it should also be mentioned that with increasing cell concentration, the solids content of the suspension increases and thus less evaporable water is sprayed. At the same spray rate, this results in higher temperatures in the process chamber and a lower moisture content of the final product. As described before, both factors have a negative influence on the survival rate, so that the described positive effect of the higher cell concentration may be greater than it seems here. This may be due to cellular aspects such as signals from quorum sensing systems in the case of high cell concentrations, which promote cell recovery and viability after drying [68].

### 3.7. Drying with Improved Formulation and Process Parameters

The previously presented results of the study of different formulation and process parameters regarding their influence in the survival of the yeast cells were used to prepare granules based on LAC, MCC and DCP with improved viabilities. Although it has already been shown that the carrier material itself has a major influence on survival, the three different materials were used again in order to be able to provide products with different formulations for the subsequent tableting step.

**Figure 8:**
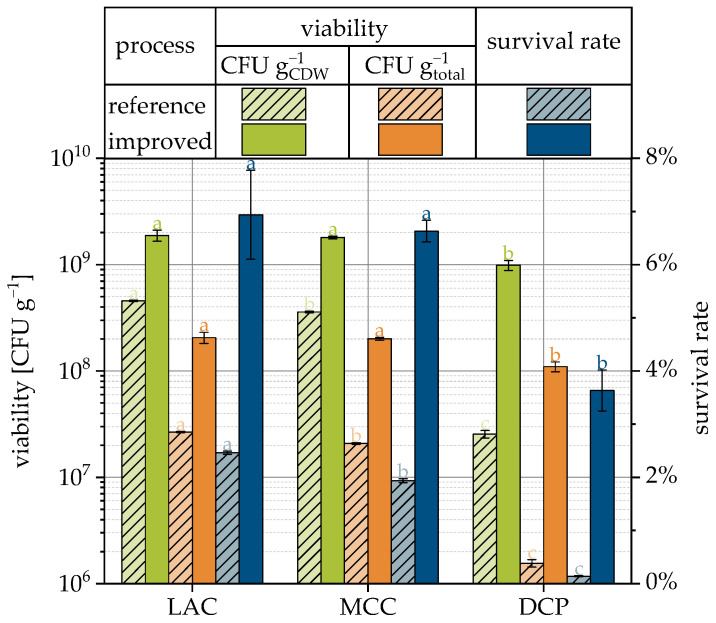
Viability related to cell dry weight and total granules weight as well as the survival rate for reference (Figure 4), and the improved process and formulation dependent on the carrier material. Data points that do not share a letter are significantly different (significance level α = 0.05).

**Figure 9:**
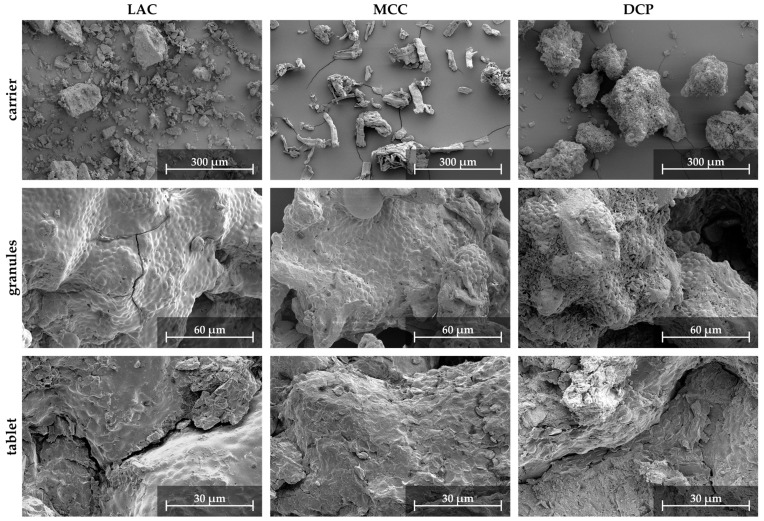
SEM images of carrier particles (**first line**), yeast granules based on the different carrier materials (**second line**), and the breaking area of tablets made at a compression stress of 400 MPa (**third line**).

The process changes resulted in increased survival rates for all three carrier materials (see Figure 8 and compare Table 3). SEM images of the granules are presented in Figure 9. The ranking of survival rates has not changed, and LAC granules still have the best survival rate and DCP granules the worst. However, the change in survival was significantly greater for DCP granules, with an increase of almost 30-fold, than for the LAC and MCC granules, each with approx. 3-fold increases. The survival rates differed correspondingly less and were approx. 6.9, 6.6 and 3.6% for LAC, MCC and DCP, respectively. The outlet temperatures were not constant during granulation, but the averaged outlet temperatures show the same order as the survival rate and were 28.4, 28.7, and 29.8 °C, respectively.

### 3.8. Tableting of Granules

**Figure 10:**
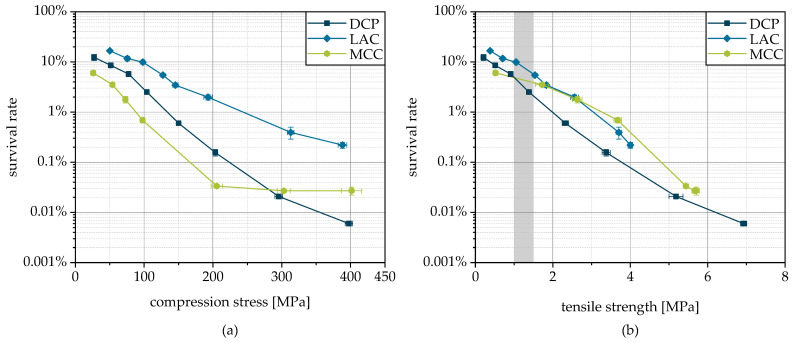
Survival of yeast cells during granulation and subsequent tableting depending on applied compression stress (**a**) and related to tablet tensile strength (**b**). The typical target strength is highlighted in gray.

For all three carrier materials used, DCP, LAC and MCC, there is a logarithmic decrease in survival with increasing compression stress (Figure 10a), although in the case of MCC, stresses above 200 MPa hardly cause a further reduction in survival. The SEM images of the breaking surfaces of tablets made from the granules, shown in Figure 9, illustrate the deformation of the cells. It is plausible that this is associated with a disruption of the cell structure resulting in a lethal loss of integrity. As expected, the formulation significantly affects the viability of the microorganisms after tableting. The highest survival at a certain compression stress was reached with LAC, followed by DCP and MCC. Thus, LAC can be stated as the best suited of the carriers tested for granulation and subsequent tableting regarding the survival of the cells. The highest survival during tableting with LAC was also found when freeze-dried yeast cells were compacted with the same excipients [9]. In the case of lyophilizates, slightly higher survival rates were possible with MCC instead of DCP. However, it should be noted that the proportion of excipients was only 20% by weight in case of the lyophilizates and approx. 80% in case of the granules. Due to this, the influence of the various excipients is also more pronounced in the case of granules. In addition, the different bulk material structures must be taken into account as they are supposed to influence the survival during compression. In the case of the tableting of comminuted lyophilizate particles, these particles contained the dried microorganisms and were mixed with the excipients as a physical mixture. Contrary to this, in the case of the granules, the excipients were present as carrier particles inside the granules, with the microorganisms bound onto the surface. This results in different stress states acting on the cells, which are linked to different degrees of mechanical damage. For example, this could influence the possible extent of the particle rearrangement during densification. Although lyophilizate particles in voids between excipient particles could be shielded from stresses, whereas the microorganisms that adhere firmly to the surface of the granule particles are subjected to intense stresses, especially at the contact points. It has been reported that the reduction in viability of microorganisms during tableting is more pronounced in the case of ductile formulations, as with MCC, due to higher shear stresses, than with brittle materials such as DCP [24]. LAC is characterized by an intermediate deformation behavior with brittle and ductile deformation fractions. Nevertheless, the survival rate curve is not between those of DCP and MCC, but at a higher level. Possibly, in the case of DCP with very high deformation resistance, higher stress peaks occur locally, which cause more stress on the cells than in case of LAC with lower deformation resistance [9]. In this context, reference should also be made to the morphology of DCP, with numerous sharp-edged crystals, which can have a detrimental effect by puncturing or slicing the cell membrane, as proposed earlier [9].

In addition to the viability of the microorganisms in the tablets, the strength of the tablets is of particular importance as it determines their handleability. Typically, a tensile strength of 1.0 to 1.5 MPa is targeted in order to ensure sufficient mechanical stability on the one hand and to prevent unnecessarily long disintegration times on the other [9]. The combined consideration of survival and tablet strength (Figure 10b) allows a direct evaluation of the suitability of the different carrier materials. Here, again, LAC is shown to be advantageous. However, the lower tabletability of these granules leads to the fact that the differences between the three formulations are not as large.

In order to take specific measures to enable increased cell survival during tableting, further studies are needed to investigate the mechanism of cell damage in more detail and to derive process-structure-property relationships that are valid across formulations. Although their transferability to probiotic microorganisms, typically of smaller size and lower mechanical strength, then needs to be verified, the derived mechanisms should contribute an important step towards a generalized understanding of microorganism damage during tableting.

## 4. Conclusions

**Figure 11:**
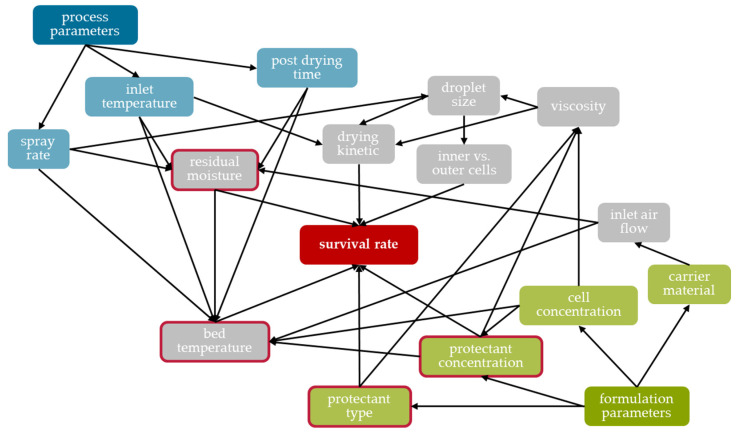
Scheme of influencing factors on the survival of microorganisms during fluidized bed granulation and their linkages. The main influences on the survival rate are highlighted with a red border.

Different formulation and process parameters were shown to influence the survival rate of *Saccharomyces cerevisiae* cells during fluidized bed spray granulation. The ability of the yeast cells to maintain their viability during this process seems to be determined by three major effects. The first is the process temperature (influenced, e.g., by inlet temperature, spray rate, inlet air volume flow, and thus, carrier material); the second is the residual moisture (influenced, e.g., by post-drying time, spray rate, inlet temperature); and the third is the effectiveness of the formulation (i.e., the added protective additives) (Figure 11). The findings from the various test series enabled the process and formulation to be rationally adapted, thus increasing the survival rate by a factor of 460 from 0.015% to as much as 6.9% (200 g of LAC as carrier, spray suspension with 200 g L^−1^ of cell dry mass and 100 g L^−1^ each of trehalose and skimmed milk powder, inlet temperature of 50 °C, spray rate of 2.64 g min^−1^, 40 min granulation time, no post-drying). A similar survival rate was achieved with MCC (6.6%), however only about half the survival rate in the case of DCP (3.6%). Nevertheless, the survival rates are low compared to other drying processes. Currently, it is assumed that this is due to the limited process window in which the fluid bed granulator used could be utilized. This concerns an over-wetting of the product and formation of lumps up to the collapse of the fluid bed if the spray rate was too high or the inlet temperature too low. Furthermore, the drying gas volume flow could not be as flexibly varied as desired. In theory, this could be increased to achieve the same drying capacity at a lower inlet temperature. However, this led to expansion of the fluidized bed via the spray nozzle, as a result of which the suspension was no longer reliably sprayed onto the particles, but also hit the vessel wall. It is believed that higher survival rates could be achieved with spray granulation if lower process temperatures could be realized; this should be considered in further studies. Nevertheless, the process has the advantage over freeze-drying in that it directly yields a tabletable product and does not require a potentially detrimental comminution step. Therefore, the subsequent tableting of the granules was investigated with regard to the damage of the yeast cells and the tensile strength of the tablets. It was possible to produce tablets with a suitable tensile strength showing an overall survival of 2 to 10% depending on the carrier material used. The increase in compression stress resulted in higher tensile strength of the tablets but caused reduced survival rates. These showed a clear dependency on the formulation, which could be attributed to the different deformation mechanisms of DCP, LAC and MCC, but also reveals the need for more detailed studies concerning the tableting of dried microorganisms. These must have the goal of deriving process models that enable the cross-formulation survival of microorganisms during compaction, and thus, a rational-based process design for tablets with probiotic microorganisms.

## Figures and Tables

**Table 1:** Overview of the process and formulation parameter combinations studied in this work. Dicalcium phosphate (DCP), lactose (LAC) and microcrystalline cellulose (MCC) were used as carrier material.

Parameter	Section 2.2.1	Section 2.2.2	Section 2.2.3	Section 2.2.4	Section 2.2.5	Section 2.2.6	Section 2.2.7
carrier material	MCC	various	LAC	LAC	MCC	MCC	various
carrier mass [g]	50	200	200	200	50	50	200
cell dry mass concentration [g L^−1^]	100	100	100	100	100	various	200
protectant(s)	various	trehalose /milk	trehalose /milk	trehalose /milk	trehalose /milk	trehalose /milk	trehalose /milk
protectant concentration [g L^−1^]	various	100/50	100/50	100/50	100/50	100/50	100/100
inlet temperature [°C]	50	50	50	various	50	50	50
spray rate [g min^−1^]	1.76	1.76	various	1.76	1.76	1.76	2.64
granulation time [min]	15	60	various	60	15	15	40
post-drying time [min]	5	10	10	10	various	5	0

**Table 2:** Particle size and density of carriers and corresponding granules. For all carrier materials, the mean particle size after granulation was significantly larger than before. Values that do not share a letter are significantly different (significance level α = 0.05).

Carrier Material	Particle Size x_50,3_ [µm]		True Density [g cm^−3^]	
	Carrier	Granules	Carrier	Granules
DCP	196 ± 8 ^a^	395 ± 5 ^A^	2.8660	2.4465
LAC	250 ± 11 ^b^	534 ± 3 ^B^	1.5366	1.5240
MCC	109 ± 2 ^c^	284 ± 5 ^C^	1.5675	1.5231

**Table 3:** Comparison of the process and formulation parameters of the initial reference processes and improved process design.

Parameter	Reference Process			Improved Process		
spray rate [mL min^−1^]	1.76			2.64		
inlet temperature [°C]	50			50		
yeast concentration [g_CDW_ L^−1^]	100			200		
trehalose concentration [g L^−1^]	100			100		
skimmed milk concentration [g L^−1^]	50			100		
post-drying [min]	10			0		
carrier mass [g]	200			200		
$\mathrm{loading}\;\left[g_{\mathrm{CDW}}{\;g}_{\mathrm{granules}}^{-1}\right]$	DCP	LAC	MCC	DCP	LAC	MCC
	0.061	0.058	0.058	0.113	0.112	0.117
particle size x_50,3_ [µm]	395 ± 5	534 ± 3	284 ± 5	622 ± 9	722 ± 1	552 ± 8
true density [g cm^−3^]	2.4465	1.5240	1.5231	2.3627	1.5152	1.5388
moisture content [%]	0 *	0.13	0.53	1.31	2.00	4.85

* No measurable mass loss occurred during drying at 80 °C for 24 h.

## Data Availability

The data presented in this study are available on request from the corresponding author. The data are not publicly available due to restrictions of the local data management systems.
